# Warming affects leaf light use efficiency and functional traits in alpine plants: evidence from a 4-year *in-situ* field experiment

**DOI:** 10.3389/fpls.2024.1353762

**Published:** 2024-03-19

**Authors:** Zijuan Zhou, Peixi Su, Jianping Yang, Rui Shi, Xinjing Ding

**Affiliations:** ^1^ Key Laboratory of Land Surface Process and Climate Change in Cold and Arid Regions, Northwest Institute of Eco-Environment and Resources, Chinese Academy of Sciences, Lanzhou, China; ^2^ School of Geography, Liaoning Normal University, Dalian, China

**Keywords:** photosynthesis, leaf traits, soil nutrients, climate change, alpine plants

## Abstract

**Introduction:**

Light use efficiency (LUE) is a crucial determinant of plant productivity, while leaf functional traits directly affect ecosystem functions. However, it remains unclear how climate warming affects LUE and leaf functional traits of dominant species in alpine meadows.

**Methods:**

We conducted a 4-year in-situ field warming experiment to investigate the eco-physiological characteristics for a dominant species (*Elymus nutans*) and a common species (*Potentilla anserina*) on the Tibetan Plateau. The leaf traits, photosynthesis and fluorescence characteristics were measured, along with the soil physical-chemical properties associated with the two species.

**Results and discussions:**

Experimental warming increased the leaf LUE, maximum photochemical efficiency, non-photochemical quenching, relative water content and specific leaf area for both species. However, there was a decrease in leaf and soil element content. Different species exhibit varying adaptability to warming. Increasing temperature significantly increased the photosynthetic rate, stomatal conductance, transpiration rate, total water content, and specific leaf volume of *E. nutans*; however, all these traits exhibited an opposite trend in *P. anserina*. Warming has a direct negative impact on leaf LUE and an indirectly enhances LUE through its effects on leaf traits. The impact of warming on plant photosynthetic capacity is primarily mediated by soil nutrients and leaf traits. These results indicate that the two different species employ distinct adaptive strategies in response to climate change, which are related to their species-specific variations. Such changes can confer an adaptive advantage for plant to cope with environmental change and potentially lead to alterations to ecosystem structure and functioning.

## 1 Introduction

The global surface temperature has risen by approximately 1.09°C since the 1850s (Masson-Delmotte et al., 2021). Climate warming is recognized as a significant driver of global change and poses a substantial threat to ecological integrity and function (Hughes et al., 2018). In the alpine ecosystem, the temperature increase has been twice as high as the global average, with a rate of 0.3-0.4°C per decade, and this trend becomes more pronounced with increasing altitude (Fu et al., 2021). Temperature is widely recognized as a major limiting factor in alpine ecosystems, and alpine vegetation exhibits a high sensitivity to temperature (Duan et al., 2019). While warming partially meets the heat requirements of plants, it also alters the micro-climate environment of plant communities, directly or indirectly impacting the photosynthetic physiological processes and consequently influencing plant growth and development (Lee et al., 2020).

Plant physiological processes are extremely sensitive to temperature, and even slight temperature fluctuations leading to significant modifications in these processes (Atkin et al., 2005). Leaf photosynthetic properties can reflect plant responses to environmental changes and play a crucial role in plant growth, thereby impacting the structure and function of ecosystems (Zhang et al., 2022). Recent research indicates that gross primary productivity is affected by photosynthesis, rather than canopy structure (Li et al., 2024). Light use efficiency (LUE) is a crucial indicator of a plant’s ability to convert absorbed light energy into chemical energy through photosynthesis (Medlyn, 1998). The strong solar radiation and long-term sunshine in the alpine region are conducive to the photosynthesis and LUE of alpine plants. However, low temperatures, significant temperature variations, short growth periods, and other factors impose limitations on plant growth and photosynthetic capacity. Warming can increase air and soil temperature, leading to a reduction in soil moisture, which in turn affects plant photosynthetic capacity and LUE (Forkel et al., 2016; Allison et al., 2018). Warming also caused a shift in plant functional traits, leading to an increase in acquisitive characteristics such as larger leaves, higher photosynthetic resource-use efficiency, thinner roots, and greater specific root length and nutrient concentrations (Wei et al., 2023). Yao et al. (2023) observed inconsistent responses in the net photosynthetic rate (*P*_n_) of sedge, grass, and shrubs under future climate scenarios. Notably, grass exhibited the least sensitivity to future temperature and CO_2_. Some studies have reported generally positive effects of climate change on plant photosynthesis, but there have also been reports of insignificant or even negative effects, which vary depending on the species-specific characteristics (Jassey and Signarbieux, 2019; Meng et al., 2023). However, it remains unclear how plant physiology will change as temperatures rise and how these changes will affect their LUE. To comprehend natural processes and ensure the long-term sustainability of alpine meadow development, it is essential to understand how physiological traits and photosynthetic capacity of alpine plants respond to climate warming.

In order to improve their survival fitness and competitive ability, plants modify their morphological and physiological characteristics in response to environmental changes and interactions with other organisms (Wang et al., 2021). A growing number of studies have demonstrated that leaf traits, such as leaf lifespan, leaf area, and leaf nutrient content, are highly sensitive to climate warming (Buzzard et al., 2019; Bjorkman et al., 2020). In both the alpine meadow and swamp ecosystems, Guittar et al. (2016) proposed that warming led to an increase in specific leaf area (SLA) for two dominant species, *E. nutans* and *C. scabrirostris*. The response of plants to climate change is also reflected in the trade-offs between resource availability and utilization. With increasing temperatures, a significant increase (Bai et al., 2013; Šímová et al., 2017) or decrease (Shen et al., 2022; Storkey and Macdonald, 2022) in leaf nitrogen content was observed. Leaf nitrogen content is consistently positively correlated with plant photosynthetic capacity due to the presence of photosynthetic enzymes and chlorophyll, which constitute a major portion of leaf nitrogen (Fu et al., 2015). The correlations between leaf traits and photosynthetic carbon assimilation are commonly employed for estimating primary production at various scales, ranging from individual leaves to global levels (Feng and Dietze, 2013).

Plant nutrient allocation reflects how plants respond to environmental changes, and the availability of soil nutrients determines the spatial and temporal patterns of leaf traits as well as plant resource utilization (Huang et al., 2021). Previous studies have indicated a close relationship between structural allocation trait (such as leaf area) and the functions of photosynthetic carbon capture, belowground nutrient acquisition, and resource transport (Kleyer et al., 2019). Changes in soil nutrients under climate warming can affect plant photosynthesis and physiological characteristics, which may further influence species composition by altering plant facilitation and competitive exclusion (Xu et al., 2022). According to Yang et al. (2022), experimental warming did not alter the content of soil organic carbon (SOC) during the growing season in an alpine meadow. Wei et al. (2023) demonstrated that plants exhibit consistent adaptive strategies in both above- and belowground traits, favoring more acquisitive traits in warmer environments. These changes could confer an adaptive advantage to plants in response to environmental change. Currently, the trade-offs between plant leaf traits and soil nutrients under climate change remain unclear, as well as the key factors that can have a greater impact on plant LUE.

Alpine meadows, as one of the typical grassland ecosystem types on the Qinghai-Tibetan Plateau (QTP), are highly sensitive to climate change (Immerzeel et al., 2020). A previous study demonstrated that the alpine meadow is becoming increasingly susceptible to the direct impacts of climate extremes, which affect ecosystem function and phenology by altering key traits of plant species (Knapp et al., 2020). The impact of global warming led to alterations in plant growth and soil nutrients in the alpine meadows. Due to low temperatures, significant diurnal temperature fluctuations, and short growth periods experienced by alpine plants, their LUE and productivity are generally very low. Therefore, gaining a better understanding of how alpine plants physiologically respond to environmental changes will greatly enhance predictions for vegetation productivity under climate change. In this study, we conducted warming experiment to investigate the effects of short-term (one year) and medium-term (four-year) warming on two alpine species in the eastern QTP. Our hypothesis was that warming could induce changes in leaf traits, chlorophyll fluorescence, and soil nutrients, thereby altering the photosynthetic potential (photosynthesis and LUE) of these plants. The specific objectives of our study were to: 1) investigate the differential impacts of short-term and medium-term warming on the physiological performances of two alpine plants; 2) elucidate the key processes that contribute to changes in leaf photosynthetic capacity under warming conditions. This research is essential for enhancing our comprehension of the species-specific differences and the potential adaptation mechanisms employed by alpine plants in response to global warming.

## 2 Materials and methods

### 2.1 Study site

The research was carried out at the Zoige Alpine Wetland Ecosystem Research Station (3440 m, 33°51′52″N, 102°08′46″E) in the eastern Tibetan Plateau. The area has a typical plateau continental semi-humid climate with no frost-free period throughout the year. The annual average air temperature is 1.1°C, and there is around 600 mm of precipitation per year, mostly during the growing season from June to September.

Based on the categorization by the US Department of Agriculture, the soil type in the research area was identified as silt clay loam, consisting of 31.2% sand, 56.0% silt, and 12.8% clay in the top 30 cm of soil. *Elymus nutans* and *Kobresia setschwanensis* are the dominant species in the study area, while other associated species include *Potentilla anserina*, *Roegneria nutans*, *Poa pratensis*, *Plantago depressa*, *Leymus secalinus*, and *Ajania tenuifolia*, etc. We choose *E. nutans* and *P. anserina* as the focal species for investigating the impacts of warming on alpine plants. The two species representing two major plant functional groups (grasses and forbs) and naturally co-exist within our study site. *Elymus nutans* is the dominant perennial grass species in the alpine meadow. *Potentilla anserina* is a common companion species and is widely distributed in alpine meadows. Due to its wide ecological amplitude and vegetative reproduction ability, *P. anserina* is considered a prime species for ecological restoration in alpine regions (Wu et al., 2022). Our field investigation at the study site found that *E. nutans* and *P. anserina* together account for nearly 60% of the total vegetation coverage. *E. nutans* and *P. anserina* display distinct stratified structures. *E. nutans* predominantly occupies the upper part of the community and grow in full-sun conditions, while the leaves of *P. anserina* were restricted to the lower parts of the community close to soil surface. Consequently, they display distinct resource utilization patterns, particularly in terms of light and soil nutrients (Zhou et al., 2021). The upper leaf layer within the canopy typically absorbs light beyond its saturation point and dissipates excess energy through heat dissipation mechanisms. In contrast, lower layer leaves often face limitations due to insufficient available light.

### 2.2 Experimental design

In April 2015, we launched our in-situ warming experiment using open-top chambers (OTCs) to assess the impacts of warming on the alpine meadow ecosystem. The OTC had a 6.4 m^2^ surface area, a height of 2 m, a bottom side length of 1.15 m, and a regular octagon form with an outside diameter of 3 m. Open areas (OAs) were developed as control regions with characteristics comparable to those of the OTCs. The detailed layout of the experimental design was mentioned in our previous articles (Zhou et al., 2021).

### 2.3 Environmental factors measurement

The ambient temperature (*T*_a_, °C) and relative humidity (*RH*, %) were measured every 30 minutes by HOBO (U23-002, Pocasset, MA, USA), which was placed in the middle of the OTCs and OAs at a height of 1.5 m above the soil surface. Using an ECH_2_O-TE sensor and EM50 data collecting system (Decagon Devices, Inc., USA), we automatically monitored soil temperature and soil moisture (volumetric soil moisture, V/V%) at a depth of 5 cm with 30-minute intervals during the experiment. Following four years of warming, the air temperature consistently increased year over year. In 2015–2018, the daily mean air temperature in the OTCs was 0.65°C, 0.74°C, 0.75°C, and 0.75°C higher than that in the OAs respectively. The average soil temperatures over the four-year period were recorded as 14.0°C in the OTCs and 13.2°C in the OAs. Warming led to a decline in soil moisture, with the OTCs experiencing reductions of 12.5%, 13.4%, 16.7%, and 10.1% during the growing seasons of 2015-2018. The lowest amount of precipitation during the growing season was recorded in 2015 (435 mm), while the highest amount was recorded in 2018 (669 mm) (see details in **Supplementary Figure S1**).

### 2.4 Gas exchange and chlorophyll fluorescence measurement

The gas exchange properties were measured using a portable photosynthetic system (LI-6400, LI-COR, Lincoln, USA). The experiments were conducted during the vigorous growth period (mid-July and mid-August) in each of the years spanning from 2015 to 2018. For comparative analysis, we selected 2015 (representing short-term warming) and 2018 (representing medium-term warming). The measurements were typically taken three times per month on clear days. Fully expanded and exposed leaves were selected, and measurements were taken from 09:00 to 13:00 with local time (which is 72 minutes later than Beijing time). For each replication, three individuals in a similar healthy state were selected. The following parameters were measured: net photosynthetic rate (*P*_n_), transpiration rate (*T*_r_), stomatal conductance (*g*_s_), stomatal limitation (*L*_s_), and intercellular CO_2_ concentration (*C*_i_). After that, the leaves were collected and their areas were scanned and precisely calculated using Image J software (version 1.47v, USA).

Leaf light use efficiency (LUE, mmol·CO_2_·mol^-1^·photons) was calculated based on the Equation 1:


(1) $$\text{LUE}={\text{P}}_{\text{n}}/{\text{PAR}}_{\text{i}}$$

Where PAR_i_ represents the incident photosynthetic active radiation (μmol·m^–2^·s^–1^).

A portable modulated chlorophyll fluorometer was used to assess the chlorophyll fluorescence characteristics immediately following photosynthesis measurements in the same plant (PAM-2100, Walz, Germany). The leaves were pre-adapted in the dark for 30 minutes before being measured hourly from 09:00-13:00 h. The maximal photochemical efficiency of PS II (*F*_v_/*F*_m_), photochemical quenching (*q*P), non-photochemical quenching (*q*N), and effective photochemical efficiency (*Yield*) of the chlorophyll fluorescence parameters were measured.

### 2.5 Leaf traits measurement

Fresh leaf samples were collected from the sunny side of each species, immediately weighed (fresh weight, FW), and then submerged in distilled water in the dark until saturated. After determining the saturated fresh weight (SW), the leaves were oven-dried at 70°C for 48 hours to estimate the dry weight (DW). The Equation 2 was used to obtain the relative water content (RWC, %) of the leaf:


(2) RWC(%)=[(FW−DW)/(SW−DW)]×100

The leaf area of the fresh leaves was measured using Image J, and specific leaf area (SLA, cm^2^·g^-1^) was calculated as the ratio of one side’s area of each leaf in each set to its dry mass (Perez-Harguindeguy et al., 2013). The drainage method was used to compute the specific leaf volume (SLV, cm^3^·g^-1^), which is the ratio of leaf volume to dry mass. Leaf dry matter content (LDMC, mg·g^-1^) was computed as the ratio of leaf dry weight to leaf saturated fresh weight. In mid-July and mid-August, three repetitions of measurements were conducted for each leaf trait index of the two species.

### 2.6 Leaf stoichiometry

After sampling, the leaves were mixed to form composite samples from ten to fifteen different individuals. The materials were crushed, sieved through an 80-mesh screen, and then dried at 70°C for 24 hours before being packed in plastic bags for measurement. A Vario Macro Cube Elemental analyzer (Elementar, Hanau, Germany) was used to assess total contents of carbon and nitrogen. Total phosphorus contents were measured using a molybdenum antimony resistance colorimetric method. For each species, the measurements were repeated three times.

### 2.7 Soil respiration and soil nutrients

The LI-8100 automatic soil CO_2_ flux system (LI-COR, Lincoln, USA) and its 20 cm survey chamber (8100-103) were used to assess soil respiration (*R*_s_). The soil collars (8100-103) were positioned with their tops 2-3 cm above the soil surface and installed one day before the measurements. The litter in the collar was cleared before each measurement, and the above-ground of the plant was subtracted.

After 24 hours of equilibration, the soil respiration rate (*R*_s_, μmol·m^-2^·s^-1^) returned to its initial level before collar insertion. Measurements were taken once per hour for two minutes between 9:00 to 13:00 with three repetitions. The *R*_s_ was computed based on the Equation 3:


(3) $$Rs=\frac{V_{C}\times P\times \frac{\partial Cs}{\partial t}}{S_{C}\times (T+273.15)\times R}$$

where *S*_C_ is the area of soil that the survey chamber covers (0.03 m^2^). *V*_C_ (m^3^) is computed as the sum of the volume of the 20 cm survey chamber (4.82×10^-3^ m^3^) and the product of the chamber offset (the distance from the settled collar to the ground inside it) and the soil area (*S*_C_), which represents the total volume of soil respiration system. $\frac{\partial {\text{C}}_{\text{s}}}{\partial \text{t}}$ is the rate of change in chamber CO_2_ during soil respiration measurements (μmol CO_2_ mol^-1^ s^-1^), where *P* is the atmospheric pressure (*P*_a_), *T* is chamber air temperature (°C), and *R* is the gas constant (8.314 Pa m^3^ mol^-1^ K^-1^).

The potassium dichromate oxidation titration was used to measure the content of soil organic carbon (SOC). The semi-trace Kjeldahl technique was used to measure the soil total nitrogen (TN), and the vanadium molybdate blue colorimetric method was employed to determine the soil total phosphorus (TP) (Xu et al., 2022). Alkaline hydrolysis was used to estimate soil available nitrogen (AN), and the molybdenum blue technique was employed to evaluate soil available phosphorus (AP) after extracting soil samples with sodium bicarbonate. After extraction with ammonium acetate, soil available potassium (AK) was measured using a flame photometric method. Using an autoanalyzer (SmartChem140, AMS Alliance, Italy), the content of soil NH_4_^+^-N was determined in extracts of 2 M KCl (1:4, soil: extractant).

### 2.8 Statistical analysis

The data was statistically analyzed using SPSS 20.0 (SPSS Inc., Chicago, USA), and the results were presented as means ± standard error (SE). Two-way ANOVA analysis was used to compare the photosynthetic physiological characteristics of two different species under varying warming treatments and years, followed by *post hoc* Duncan multiple comparison for further analysis. Principal component analysis (PCA) was employed to compare the variance in leaf traits among species and treatments over the course of four years. The plspm package in structural equation modeling (SEM) was utilized to investigate the influence of leaf functional traits and environmental factors on leaf photosynthetic capacity. The analyses were conducted using R program v3.4.4, and figures were generated with Origin Pro 2021 (OriginLab Corporation, United States).

## 3 Results

### 3.1 Leaf physiological and biochemical characteristics

#### 3.1.1 Leaf photosynthesis and light use efficiency

**Figure 1:**
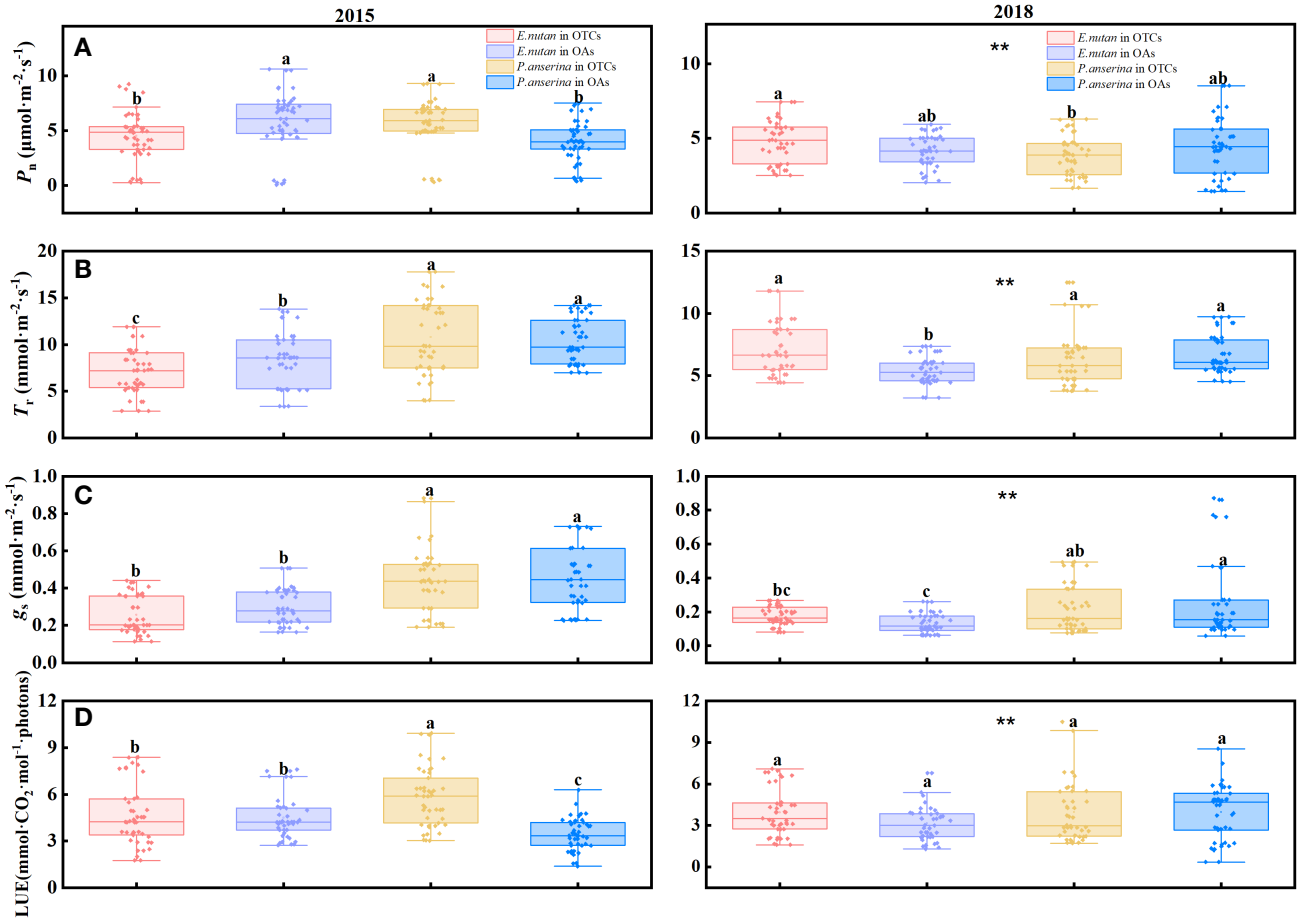
Effects of warming on photosynthesis and light use efficiency of *E nutans* and *P. anserina* in different years. The values are presented as means ± SE, with different letters indicating significant differences among species in the same year under warming at *p<* 0.05. ** indicates significant differences between different years. **(A)** net photosynthetic rate, **(B)** transpiration rate, **(C)** stomatal conductance, **(D)** light use efficiency. (The comparison of differences between species, treatments and years is shown in **Supplementary Table S1**).

**Figure 2:**
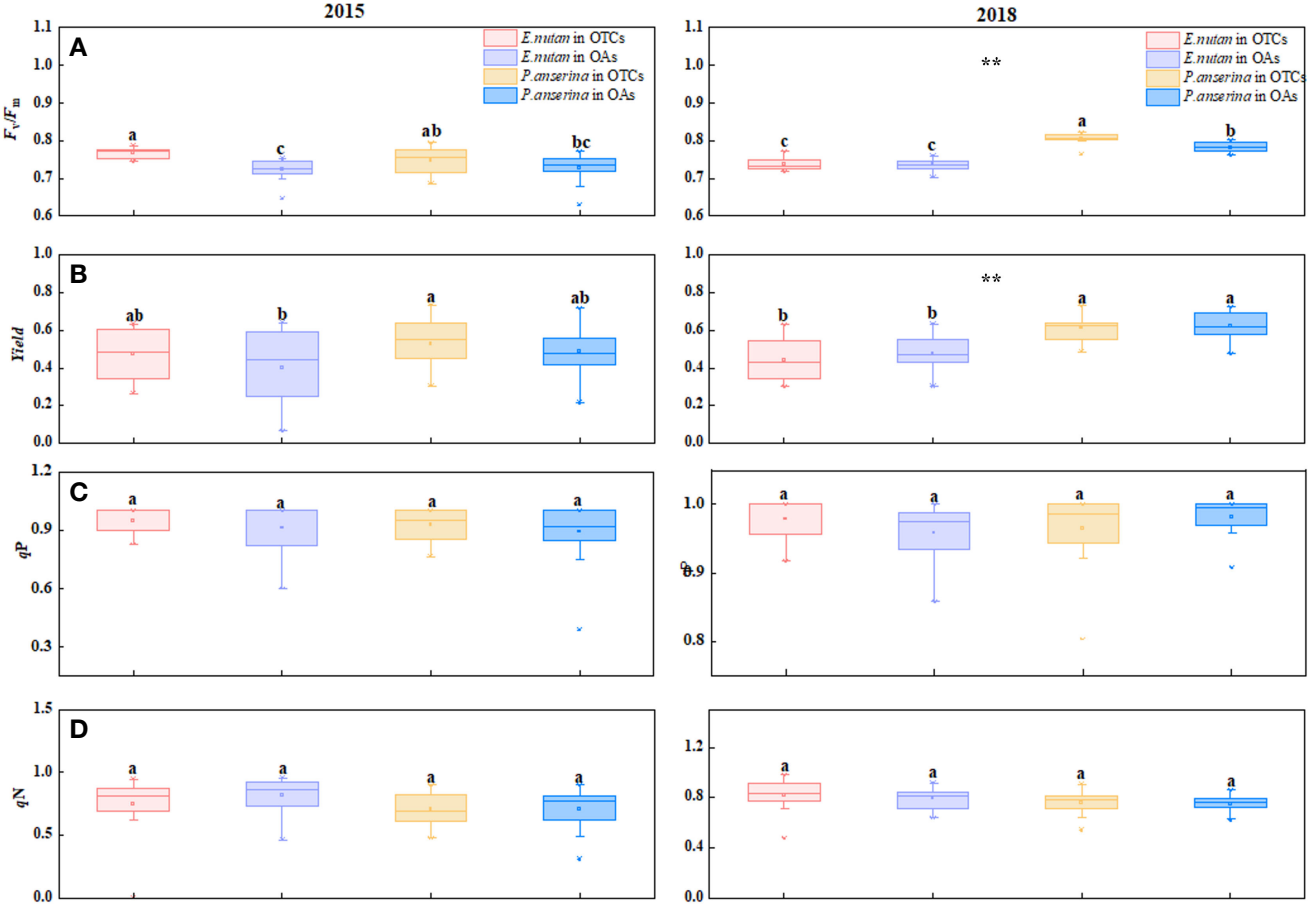
Effect of warming on chlorophyll fluorescence characteristics of *E nutans* and *P. anserina* in different years. The values are presented as means ± SE, with different letters indicating significant differences among species in the same year under warming at *p<* 0.05. ** indicates significant differences between different years. **(A)** maximum photochemical efficiency of PSII (*F*_v_/*F*_m_), **(B)** effective photochemical efficiency (*yield*), **(C)** photochemical quenching (*q*P), **(D)** non-photochemical quenching (*q*N). (The comparison of differences between species, treatments and years is shown in **Supplementary Table S1**).

Different warming years had significant impacts on the photosynthesis rate (*P*_n_) and transpiration rate (*T*_r_) of both species. Specifically, short-term warming in 2015 resulted in decreased *P*_n_ and *T*_r_ for *E. nutans*, while increasing *P*_n_ and *T*_r_ for *P. anserina*. Conversely, medium-term warming in 2018 led to increased *P*_n_ and *T*_r_ for *E. nutans* but decreased for *P. anserina* (**Figures 1A**, **2B**). Significant differences in *P*_n_ and *T*_r_ were observed between the two species. When comparing the *P*_n_ of both species in 2015 and 2018 under warming conditions, it was found that the *P*_n_ of both species decreased with the prolonged duration of warming.

In short and medium-term warming, the stomatal conductance (*g*_s_) of *P. anserina* was significantly higher than that of *E. nutans* (**Figure 1C**). There were significant differences in *g*_s_ between *E. nutans* and *P. anserina* across different years of warming. In 2015, both *E. nutans* and *P. anserina* experienced a reduction in *g*_s_ due to warming, while in 2018, the *g*_s_ of *E. nutans* increased while that of *P. anserina* decreased.

The LUE of *E. nutans* and *P. anserina* exhibited an increase with warming in the short and medium-terms (**Figure 1D**). In 2015, the LUE of *E. nutans* was measured at 4.64 and 4.49 mmol·CO_2_·mol^-1^·photons in the OTCs and OAs, respectively, while the LUE of *P. anserina* was recorded as 5.80 and 3.43 mmol·CO_2_·mol^-1^·photons in the OTCs and OAs. In 2018, warming increased the LUE of both *E. nutans* and *P. anserina*. However, there was no statistically significant difference in LUE between the two species (*p* > 0.05).

#### 3.1.2 Chlorophyll fluorescence characteristics

In both 2015 and 2018, warming increased the *F*_v_/*F*_m_ values of both species (**Figure 2A**). In 2015, the *F*_v_/*F*_m_ values of *E. nutans* were 0.776 and 0.723 in OTCs and OAs, respectively, showing a statistically significant difference (*p<* 0.05). The *F*_v_/*F*_m_ values of *P. anserina* also increased with warming, but the difference was not statistically significant (*p* > 0.05). In 2018, the *F*_v_/*F*_m_ value of *P. anserina* was higher than that of *E. nutans*. The *yield* values of the two species exhibited opposite trends under short-term and medium-term warming, with *P. anserina* having a higher *yield* than *E. nutans*, and the *yield* values of the two plants exhibited significant differences (**Figure 2B**). Under short-term warming, the *yield* of *E. nutans* and *P. anserina* increased, while in medium-term warming, the *yield* of both species decreased.

The *qP* values of the two species exhibited significant differences across different warming years (*p<* 0.05). In 2015, warming increased the *qP* values of both species, with *E. nutans* exhibiting a higher value compared to *P. anserina* (**Figure 2C**). In 2018, warming increased the *qP* values of *E. nutans* and decreased those of *P. anserina*, but the difference was not statistically significant (*p* > 0.05). Short-term warming (2015) reduced the *qN* values of both *E. nutans* and *P. anserina*, while medium-term warming increased them (**Figure 2D**).

#### 3.1.3 Leaf physiological traits

Regarding the evaluated leaf physiological parameters, *E. nutans* and *P. anserina* responded differentially to in-situ warming (**Table 1**). *E. nutans* and *P. anserina* had significant differences in the contents of TWC, SLA, SLV, and LDMC under different warming treatments (*p<* 0.05). During the vigorous growth periods (July and August) in 2015, there were significant changes in the leaf physiological parameters of *P. anserina*, while those of *E. nutans* remained relatively stable. Warming increased the contents of TWC, SLA, and SLV in *E. nutans*, and reduced the contents of TWC, RWC, SLA, and SLV in *P. anserina*. Different trends were observed in *E. nutans* and *P. anserina* under medium-term warming (2018). The TWC and SLV contents of *E. nutans* increased under in-situ warming, while decreased in *P. anserina*. Meanwhile, the RWC and SLA contents of *E. nutans* and *P. anserina* both increased. The content of LDMC decreased in *E. nutans* and increased in *P. anserina* in the OTCs.

**Table 1:** Changes in leaf traits of *E. nutans* and *P. anserina* under simulated warming in different years.

	species	experiments	TWC (%)	RWC (%)	LDMC (mg·g^-1^)	SLA (cm^2^·g^-1^)	SLV (cm^3^·g^-1^)
2015	*E.nutans*	OTCs	60.9 ± 1.6 b	65.8 ± 4.3 b	294.6 ± 7.1 b	197.8 ± 4.4 a	5.26 ± 0.6 b
		OAs	60.8 ± 1.0 b	71.7 ± 3.1 ab	315.3 ± 7.6 b	189.5 ± 5.9 a	5.13 ± 0.3 b
	*P. anserina*	OTCs	60.9 ± 2.1 b	66.5 ± 5.7 b	294.0 ± 9.0 b	127.0 ± 7.2 b	5.11 ± 0.7 b
		OAs	69.1 ± 1.6 a	82.7 ± 2.1 a	269.8 ± 15.5 c	178.7 ± 4.8 a	7.35 ± 0.7 a
2018	*E.nutans*	OTCs	64.6 ± 2.0 a	81.7 ± 3.7 a	308.7 ± 21.1 b	187.1 ± 12.9 a	4.95 ± 0.3 a
		OAs	59.5 ± 4.4 a	79.9 ± 3.6 a	351.0 ± 34.6 a	179.9 ± 17.4 a	4.43 ± 0.4 a
	*P. anserina*	OTCs	60.5 ± 0.6 a	79.8 ± 3.9 a	341.3 ± 8.2 a	152.6 ± 14.7 b	4.47 ± 0.5 a
		OAs	62.9 ± 0.9 a	78.4 ± 1.3 a	316.1 ± 8.8 b	149.9 ± 11.5 b	5.38 ± 0.5 a
Significance	years		ns	**	**	ns	**
	species*years		ns	ns	ns	ns	ns
	years* treatments		ns	**	ns	ns	ns
	years*species* treatments		ns	ns	ns	ns	ns

Values are presented as means ± SE (n=6). Values with the same lowercase within columns indicate no significant differences among species in the same year under warming at *p*< 0.05. ** indicates significant differences among different years, species and treatments at the level of *p*< 0.05, and ns indicates not significant. TWC, the total water content; RWC, relative water content; LDMC, leaf dry matter content; SLA, specific leaf area; SLV, specific leaf volume.

#### 3.1.4 Leaf element content

**Figure 3:**
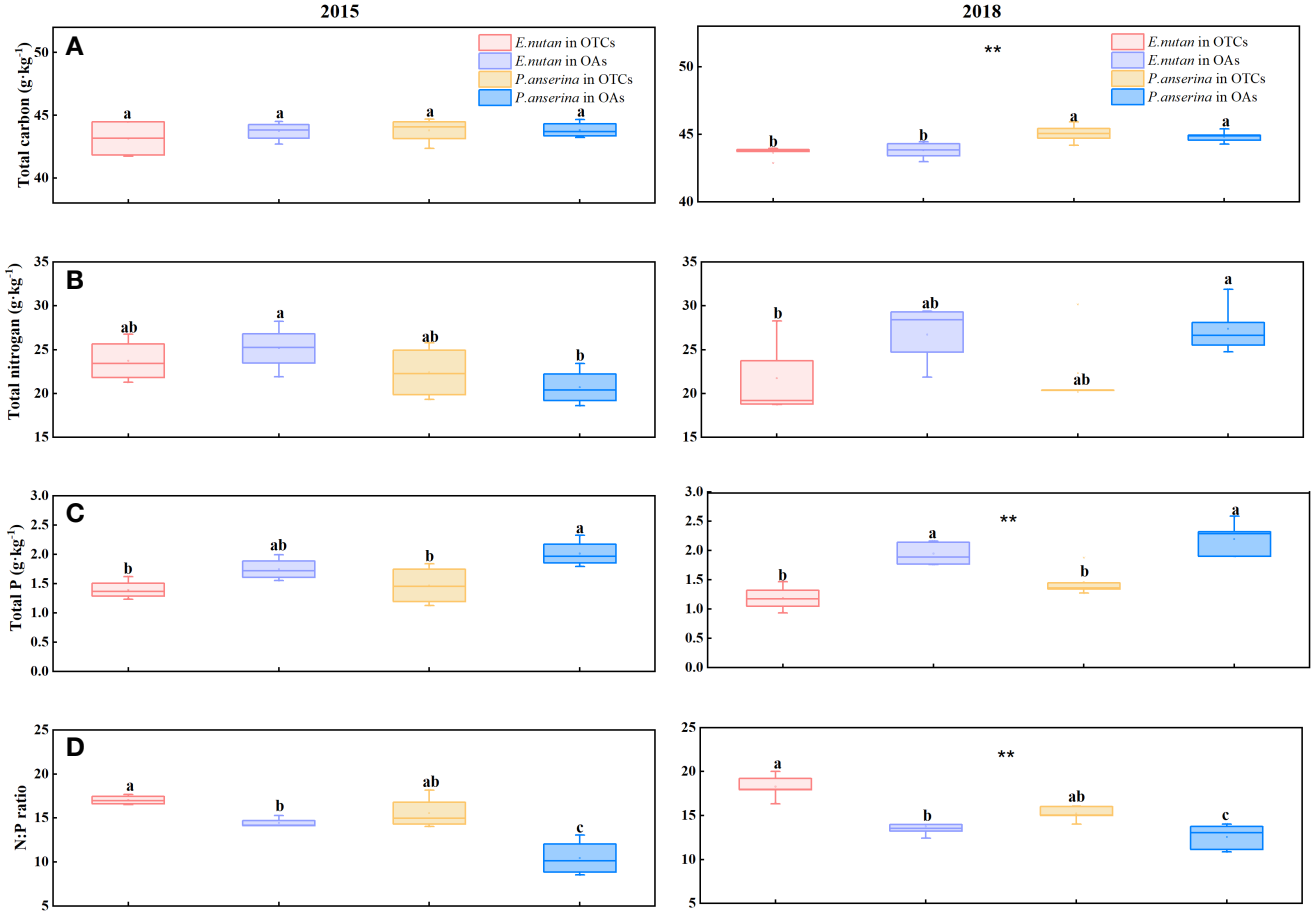
Effects of warming on the element content in *E. nutans* and *P. anserina* in different years. The values are presented as means ± SE, with different letters indicating significant differences among species in the same year under warming at *p<* 0.05. ** indicates significant differences between different years. **(A)** total carbon content, **(B)** total nitrogen content, **(C)** total phosphorus contents, **(D)** the N:P ratios. (The comparison of differences between species, treatments and years is shown in **Supplementary Table S1**).

In both 2015 and 2018, *P. anserina* exhibited a higher total carbon (C) content compared to *E. nutans*, and there are significant differences between both two species (*p<* 0.05). In 2015, warming led to a decrease in the total carbon content of both species; however, in 2018, there was an increase in the total carbon content of *P. anserina* and a decrease in that of *E. nutans* (**Figure 3A**). Short-term warming decreased the total nitrogen (N) content of *E. nutans* while increased the total N content of *P. anserina*; however, the total N content of *E. nutans* was higher than that of *P. anserina* (**Figure 3B**). The total N content of the two species decreased under medium-term warming. The total phosphorus (P) content of the two species decreased significantly due to warming in the short and medium-terms (**Figure 3C**), while the leaf N:P ratio showed an opposite trend. The N:P ratios exhibited significant variations between the two species exposed to different warming treatments (**Figure 3D**). Under different treatments of short and medium-term warming, the N:P ratio of the two species experienced a remarkable increase. In 2015, the N:P ratios of *E. nutans* were 17.04 in OTCs and 14.42 in OAs, respectively, while those of *P. anserina* were 15.54 in OTCs and 10.46 in OAs. In 2018, the N:P ratios of *E. nutans* were 18.30 in OTCs and 13.75 in OAs, respectively, while those of *P. anserina* were 15.23 in OTCs and 12.57 in OAs.

### 3.2 Soil physicochemical properties

#### 3.2.1 Soil respiration

**Figure 4:**
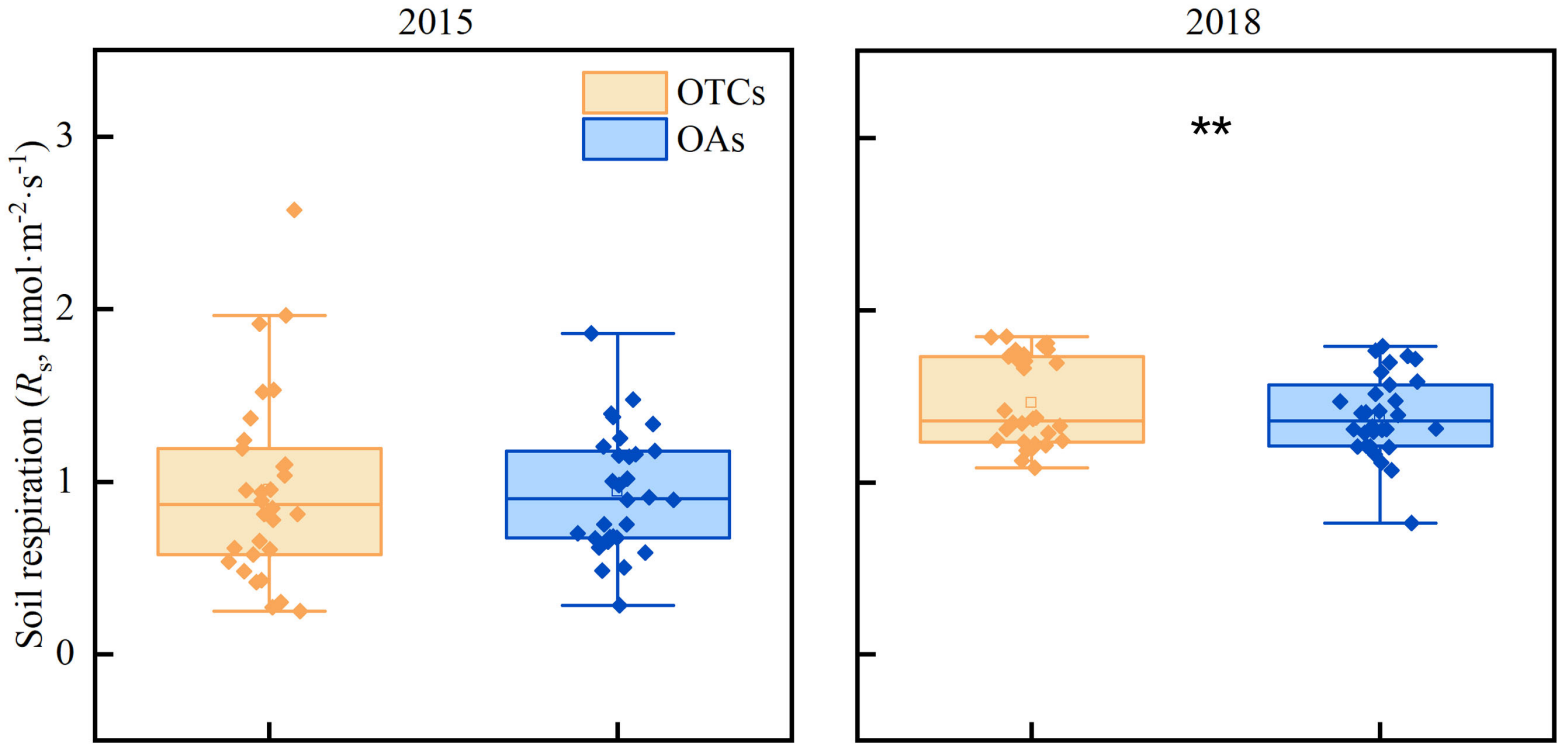
The effect of *in-situ* warming on soil respiration (*R*_s_) in 2015 and 2018. ** indicates significant differences among different years at the level of *p*< 0.05.

It has been observed that the gradual increase in temperature has led to a slight rise in soil respiration (*R*_s_) (**Figure 4**). In 2015, the *R*_s_ of OTCs and OAs were recorded as 1.06 and 0.97 μmol·m^-2^·s^-1^ respectively, while in 2018, the *R*_s_ of OTCs and OAs increased to 1.46 and 1.39 μmol·m^-2^·s^-1^ respectively. The impact of warming on *R*_s_ varied significantly across different years (*p*< 0.05), but there was minimal disparity between the various treatments (OTCs *vs*. OAs, *p* > 0.05).

#### 3.2.2 Soil element content

Short-term and medium-term warming led to a decrease in the content of soil organic carbon (SOC), soil total nitrogen, soil total phosphorus, soil available phosphorus, soil available potassium, and soil NH_4_^+^-N (**Table 2**). In the short-term warming, there was a significant decline in soil available potassium and NH_4_^+^-N contents, while in the medium-term warming, there was a significant decrease in soil total phosphorus, and available phosphorus contents (*p<* 0.05). The total C content increased significantly in both 2015 and 2018 (*p<* 0.05).

**Table 2:** Changes in soil element content under simulated warming in 2015 and 2018.

	experiments	SOC (g kg ^-1^)	TC (g kg ^-1^)	TN (g kg ^-1^)	TP (g kg ^-1^)	AP (mg kg ^-1^)	AK (mg kg ^-1^)	NH_4_^+^-N (mg kg ^-1^)
2015	OTCs	28.17 ± 2.1	71.51 ± 1.1	2.74 ± 0.2	1.44 ± 0.1	4.45 ± 1.3	128.6 ± 10.6	170.5 ± 21.4
	OAs	30.26 ± 1.3	54.02 ± 1.4	3.09 ± 0.1	1.63 ± 0.1	6.21 ± 1.0	315.7 ± 17.2	249.1 ± 7.7
2018	OTCs	29.34 ± 1.0	67.77 ± 1.0	3.30 ± 0.1	1.31 ± 0.1	8.57 ± 0.8	170.0 ± 17.8	221.9 ± 12.4
	OAs	32.92 ± 2.4	63.22 ± 1.8	3.63 ± 0.3 a	2.04 ± 0.2	16.48 ± 2.7	218.9 ± 22.1	248.4 ± 16.9
significance	year	ns	ns	**	ns	**	ns	ns
	treats	ns	**	ns	ns	**	**	**
	year* treats	ns	**	ns	ns	ns	**	ns

Values are presented as means ± SE (n=6). ** indicates significant differences among different years and treatments at the level of *p*< 0.05, and ns indicates not significant. SOC, soil organic carbon; TC, total carbon; TN, total nitrogen; TP, total phosphorus; AP, soil available phosphorus; AK, soil available potassium. NH_4_^+^-N, soil ammonium nitrogen.

### 3.3 Main factors affect leaf photosynthetic capacity under warming

**Figure 5:**
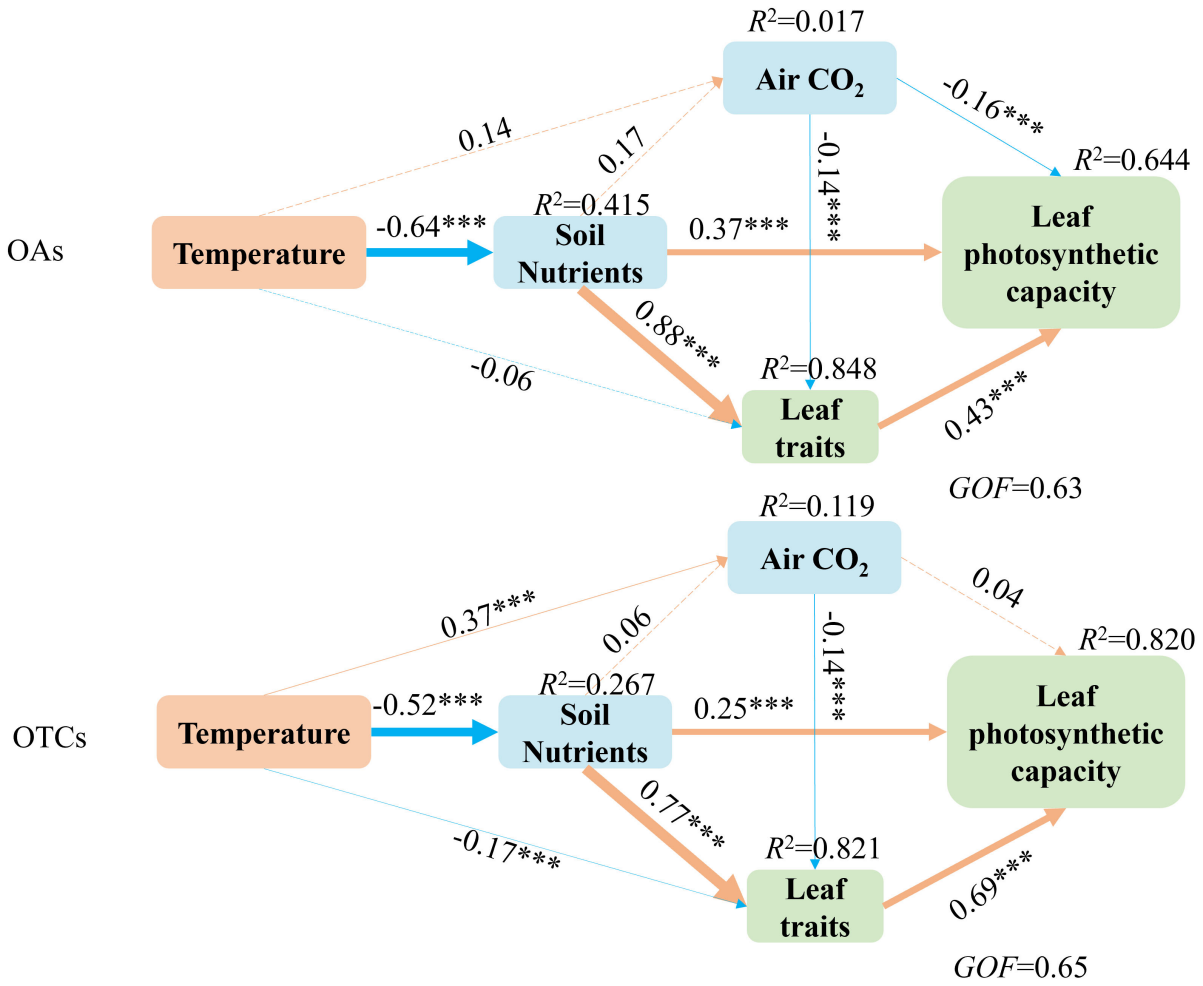
Structural equation models reveal direct and indirect influences of soil nutrients, atmospheric CO_2_, leaf traits, and leaf photosynthetic capacity on warming. Single-arrowed pathways indicate the directional effect between variables. The values associated with pathways are the standardized path coefficients. The *R* ^2^-values are provided for soil nutrients, atmospheric CO_2_, leaf traits, and leaf photosynthetic capacity to indicate the variance explained by the model (*R^2^*). The width of the arrows indicates the strength of the relationships. Orange arrows indicate significant positive relationships, while blue arrows indicate significant negative relationships. The numbers on the line represent standardized path coefficients, and stars indicate significant correlations. ***p< 0.001.

The data representing the interaction pathways between soil nutrients, atmospheric CO_2_, leaf traits, and leaf photosynthetic capability in response to warming was adequately fit by structural equation models (SEM). It can be seen that soil nutrients have significant effects on leaf traits both in OTCs and OAs. The SEM analysis revealed that warming impacts leaf photosynthetic capability indirectly through soil nutrients, atmospheric CO_2_, and leaf traits in both OAs and OTCs. Firstly, the temperature had a significant effect on atmospheric CO_2_ and leaf traits in OTCs but not in OAs. Secondly, the photosynthetic capability of OTCs was more affected by temperature variations than that of OAs. Thirdly, the effects of temperature variation on leaf traits were greater in OTCs than in OAs. Furthermore, the correlation between leaf traits and photosynthetic capacity increased in OTCs, while the correlation between soil nutrients and photosynthetic capacity decreased (**Figure 5**). Finally, atmospheric CO_2_ had a significant effect on leaf photosynthetic capacity in OAs but not in OTCs, indicating that the effect of atmospheric CO_2_ on leaf photosynthetic capacity decreased with increasing temperature.

A PCA-Biplot was used to compare the variance in leaf traits among different species and treatments over a period of four years. The first and second PC axes explained 27.3% and 20.1%, respectively. For the two species, *E. nutans* showed a strong correlation with *L*_s_, SLA, RWC, TN, TC, NP, *qN*, *Yield*, *F*_v_/*F*_m_ and LDMC. *P. anserina* exhibited a strong correlation with *P*_n_, *G*_s_, *T*_r_, LUE, SLV, TWC and TP (see the **Supplementary Figure S2A**). The warming treatment (OTCs) had a significant impact on the photosynthetic capability (photosynthesis and fluorescence) of species (see the **Supplementary Figure S2B**).

## 4 Discussion

### 4.1 Responses of leaf photosynthetic and LUE to climate warming

Alpine plants are largely restricted by low temperatures, and warming might directly reduce the impact of low temperatures on plant growth, even change the community structure and species composition of alpine meadows (Li et al., 2018; Zhou et al., 2021). Photosynthesis can reflect the physiological adaptability of plants under specific environmental conditions. Several studies suggest that alpine plants exhibit higher photosynthetic capacities and leaf nitrogen concentrations compared to the global average (Wright et al., 2004; He et al., 2006). The majority research has shown that warming has positive effects on plant photosynthesis. Fu et al. (2015) discovered that warming significantly increased in the *P*_n_ of the alpine plants on the Tibetan Plateau, which was related to the increase of stomatal conductance (*g*_s_), chlorophyll (*Chl*) content, *yield*, and non-photochemical quenching of *Chl* fluorescence. Carroll et al. (2017) found that the photosynthesis of three dominant species (*Pinus contorta*, *P. ponderosa*, and *Populus tremuloides*) had different responses to warming. Climatic warming also affects leaf photosynthetic physiological parameters, such as *Chl* fluorescence, *g*_s_, and intercellular CO_2_ concentration (*C*_i_), which are all temperature-dependent (Ruiz-Vera et al., 2013). In our research, higher *g*_s_ was linked to better photosynthetic carbon absorption capability. The *g*_s_ affects CO_2_ diffusion from the atmosphere into the intercellular space of leaves, and high *g*_s_ promotes plant photosynthesis and C assimilation (Sugiura et al., 2020). Moreover, we observed a significant difference in the *g*_s_ of both species between the two durations of warming. Specifically, compared to the short-term warming, the two species exhibited a decrease in *g*_s_ during the medium-term warming, which can be attributed to a simultaneous decrease in *P*_n_.

Photosynthesis provides a comprehensive depiction of a plant’s physiological condition, which can be used to quantify growth differences between plants and the degree of environmental impact (Lin et al., 2017). The response of photosynthesis may differ between short-term and medium-term warming. Under short-term warming, the *P*_n_ of *E. nutans* decreased while that of *P. anserina* increased. However, the *P*_n_ of the two species had an opposite trend during medium-term warming. The *P*_n_ of *E. nutans* increased, while that of *P. anserina* decreased. Phenotypic plasticity is widely recognized as a primary mechanism by which plants adapt to variations in environmental factors, serving as an observed adaptation to short-term fluctuations in the environment (Gratani, 2014). The contrasting effects of short-term warming on *P*_n_ and *T*_r_ for *E. mutans* versus *P. anserina*, possibly attributed to phenotypic plasticity rather than adaptation. In the alpine meadow, *E. nutans* was the dominant species, while *P. anserina* was a common subordinate species. The height of a plant plays a crucial role in determining its ability to compete for light. Both of these two species have a distinct layered structure, and their photosynthetic capacity and LUE are significantly different. The competitive coexistence of different functional groups in alpine meadows is primarily attributed to their disparities in canopy photosynthetically active radiation, soil nutrient acquisition (Kleyer et al., 2019; Rao et al., 2023). Climate warming has resulted in a decline in soil nutrient and moisture, intensifying the competition between *E. nutans* and *P. anserina*. Consequently, this competition has led to changes in their leaf traits, ultimately impacting their photosynthetic capacity. In addition, climate change can directly influence plant photosynthesis through alterations in temperature and precipitation patterns. Ma et al. (2017) suggested that the two dominant species, *E. nutans* and *Stipa aliena*, were relatively insensitive to environmental changes, probably because of their greater ability to acquire nutrients and light. Warming increased the photoinhibition of *E. nutans* but decreased the photoinhibition of *P. anserina*, which is consistent with previous researches (Shi et al., 2010; Zhou et al., 2021).

The *F*_v_/*F*_m_ value is a sensitive indicator of photoinhibition. In our study, the *F*_v_/*F*_m_ value of the two species in the OTCs was higher than that of the OAs, indicating that the alpine plants were restricted by low temperatures, and warming improved their ability to resist stress (**Figure 2**). Under medium-term warming, the *F*_v_/*F*_m_ value of *P. anserina* was higher than that of *E. nutans*, indicating more effective carboxylation and quicker light-harvesting by the PSII antenna complexes. Changes in *F*_v_/*F*_m_ value reflect up-or down-regulation of PSII, which is associated with changes in *q*P or *q*N in PSII (Wu et al., 2018). *q*N is an indicator of a plant’s ability to reduce heat dissipation in its photosynthetic membranes, thereby minimizing chloroplast damage (Ware et al., 2015). *E. nutans* in the OTCs activated photoprotection and reduced photoinhibition mechanisms, resulting in a greater *q*N to dissipate excess heat and sustain C assimilation capacity.

Understanding the LUE of alpine plants is crucial for enhancing their productivity and mitigating the degradation of alpine meadows. Zhang et al. (2015) believed that the changes in plant height and coverage under warming directly affect their competition for light energy and LUE. Our study revealed that warming enhanced the LUE of both two species, with significant variations observed among different species and over different years of warming (**Figure 1**). In contrast to our findings, Zhou et al. (2016) suggest that simulated warming may reduce the LUE of alpine meadows due to the negative impact of warming-induced dry micro-environment on LUE, which masks the favorable effect of temperature rises. Overall, our results imply that both of the two species were impacted by climate change and that the short and medium-term impacts on various species varied. Warming improved the LUE of the two species, which was beneficial to the growth and productivity of alpine plants.

### 4.2 Response of leaf traits to climate warming

Leaf traits are important indicators of plant adaptation to environmental change because they are linked to the efficiency of plant resource acquisition and use (Li et al., 2018; Xu et al., 2018; Li et al., 2021). In addition, leaf traits account for the majority of the variation in ecosystem productivity (Sigdel et al., 2023). Numerous studies have demonstrated that leaf traits, such as leaf lifespan, leaf area, and leaf nutrient content, are highly sensitive to climate warming (Myers-Smith et al., 2019; Bjorkman et al., 2020). Warming directly affects leaf traits and indirectly affects plant photosynthesis and LUE through these traits. Our research indicate that the impact of warming on leaf photosynthetic capacity varies among the two species, which is related to their functional traits and soil nutrient availability (**Figure 5**). Under medium-term warming, both species’ RWC and SLA increased. Additionally, the TWC and SLV of *E. nutans* increased and the LDMC of *P. anserina* also increased. Our findings support the hypothesis that phenotypic plasticity in certain plant traits can serve as a predictor for community performance under climate change.

SLA and leaf nitrogen content play a crucial role in carbon fixation (Díaz et al., 2016). According to Kattge et al. (2020), SLA exhibits sensitive to climate change and is closely associated with species-specific resource utilization. It plays a crucial role in influencing photosynthesis, light interception, and plant growth, while also serving as a predictor of competitiveness and environmental tolerance (Worthy et al., 2020). Plants with a larger leaf area are more efficient in capturing light and carbon. Our research showed that the SLA of the dominant species *E. nutans* is enhanced by warming, which implies higher resource acquisition in warmer climates. This finding is consistent with many other studies (Guittar et al., 2016; Liu et al., 2018). SLV is an important leaf functional trait, is influenced by factors such as leaf thickness, overall dimension, and dry matter content. It serves as an indicator of a plant’s ability to adapt to extreme environments like cold and arid conditions (Su et al., 2018). Leaf volume is determined by the combination of photosynthetic area and thickness, representing the entirety of photosynthetic organs. This trait facilitates better comparison among different plant species. Su et al. (2018) proposed the concept of SLV and suggested that alpine plants with higher SLV would exhibit greater resistance to harsh environmental conditions. In our study, we observed a strong positive correlation between leaf SLV and leaf *P*_n_ during the medium-term warming (**Figure 5**). The higher the leaf *P*_n_ of plants, the greater the SLV; however, short-term warming exhibited an opposite trend. These results indicate that during the process of long-term adaptation to the environment, the photosynthetic capacity of plants and leaf traits are mutually adapted and coordinated.

Leaf nitrogen content is strongly correlated with photosynthetic capacity, as nitrogen is essential for the synthesis of Rubisco, which is the key enzyme in photosynthesis (Reich et al., 1994). We observed a positive correlation between leaf C and N content and LUE. Peng et al. (2020) suggested that warming slightly increased the coverage of legumes and the C: N ratio of all plants in the alpine meadow. Our investigation revealed that the C: N and N: P ratios of the two species’ leaves increased under warming, which is consistent with Peng’s findings. The alpine meadow subjected to experimental warming displayed higher leaf C: N and N: P ratios, indicating that plants were more efficient in utilizing nitrogen for growth. Plant communities exhibit both positive and negative interactions between different plant species (Callaway and Walker, 1997). Cui et al. (2023) confirmed that grasses exhibited a higher competitive ability compared to other functional groups, primarily attributed to their increased investment in roots and enhanced capacity for resource uptake.

### 4.3 Effects of environmental factors on light use efficiency of plants

The productivity of grasslands is influenced by the interactions between soil and climatic conditions (Shen et al., 2022). Our structural equation modeling revealed that warming indirectly impacts leaf photosynthetic capacity through factors such as soil nutrients, atmospheric CO_2_, and leaf traits (**Figure 5**). Our previous studies have demonstrated that the optimal temperature range for alpine plants is between 20°C and 25°C (Zhou et al., 2017). During the vigorous growth period in 2015 and 2018, between 09:00 and 13:00 hours, we used an LI-6400 portable photosynthesis system to measure average air temperatures of approximately 28.5°C and 31.5°C. These temperatures exceeded the optimal range for alpine plants, resulting in a negative impact on leaf photosynthetic capacity.

Soil plays a crucial role in providing the majority of nutrients necessary for plant growth, and these nutrients are closely associated with how plant leaves utilize resources (Gao et al., 2019). The soil nutrients (N) have a significant impact on leaf N and plant photosynthesis (Yu et al., 2019), and a low soil phosphorus (P) may result in reduced leaf P content, thereby limiting overall plant function (Sun et al., 2019). Climate change modifies the physical and chemical properties of soil, thereby impacting the functioning of the alpine meadow ecosystem. Our findings indicate that short and medium-term experimental warming have similar impacts on soil nutrients. Warming reduced soil nutrient contents at depths of 0-30 cm (**Table 2**), indicating that warming stimulated soil nutrient cycling and organic matter decomposition, resulting in a decrease in soil C and N contents (Xu et al., 2022). However, some studies have shown inconsistent results regarding the responses of soil nutrients to climate warming. While some suggest that warming has no impact on soil C and N contents (Ning et al., 2019; Yang et al., 2022), others indicate an increase (White-Monsant et al., 2017). These contradictory results may be attributed to variations in temperature and duration of warming, as well as the diversity of grassland ecosystems. In our study, soil nutrient content did not change significantly under warming. This is because, in the case of relatively short-term warming experiments (< 5 years), it was challenging to significantly alter the vast soil carbon pool due to the substantial spatial heterogeneity in soil organic carbon among plots and the limited number of repeated warming experiments.

Several studies indicate a positive correlation between warming-induced changes in plant total biomass, above-ground biomass, and below-ground biomass with soil nutrient content. This subsequently impacts the LUE of plants (Song et al., 2019; Zhou et al., 2022). Shen et al. (2022) found that the change in soil pH and nutrient imbalance caused by N and P enrichment were the main factors impacting the photosynthetic characteristics of plant in the alpine steppe. In our study, the effects of soil nutrients on LUE were primarily mediated through their impact on leaf traits. The content of soil nitrogen significantly influences leaf N content and plant photosynthesis, while inadequate levels of soil P can lead to reduced leaf P content and limit leaf function (Gong and Gao, 2019; Sun et al., 2019).

Soil respiration (*R*_s_) is expected to have positive feedback on global warming (Wang et al., 2021). Some studies suggest that short-term warming promotes soil respiration, but there is no consistent pattern under long-term warming and variations exist among ecosystems (Chen et al., 2016; Ganjurjav et al., 2018). According to García-Palacios et al. (2021), *R*_s_ is highly sensitive to temperature, and warming will stimulate *R*_s_ activation and accelerate its rate. Yu et al. (2019) found no significant effects of experimental warming on *R*_s_. Our findings were similar to prior research that alpine meadow *R*_s_ increased in response to both short- and medium-term warming.

## 5 Conclusions

In this study, we conducted a 4-year in-situ field experiment to investigate the effects of warming on photosynthesis and LUE of alpine plants on the Tibetan Plateau. Our findings suggest that the two typical alpine species were affected by climate warming, and the effects of short and medium-term warming on different species exhibited significant species-specific variations. Warming improved the LUE of both species, which was beneficial to the growth and productivity of alpine plants. During the medium-term adaptation to the environment, the photosynthetic capacity of plants and leaf traits are mutually adapted and coordinated. Leaf traits, such as SLV, showed a significant positive correlation with leaf *P*_n_. Additionally, warming primarily enhances leaf functional traits by altering soil nutrients and thus affects leaf photosynthetic capacity. Our findings will be useful in understanding the underlying mechanisms of alpine plant responses to global warming.

## Data availability statement

The original contributions presented in the study are included in the article/**Supplementary Material**, further inquiries can be directed to the corresponding author/s.

## Author contributions

ZZ: Writing – original draft, Writing – review & editing. PS: Supervision, Writing – review & editing. JY: Writing – review & editing, Visualization, Data curation. RS: Writing – review & editing, Investigation. XD: Writing – review & editing, Investigation.

## Supplementary material

Supplementary Figure 1 Air temperature at a height of 1.5 m (°C), soil temperature at 5 cm depth (°C), soil moisture at 5 cm depth (%), and daily precipitation (mm) during the growing season (from May to September) in open-top chambers (OTCs) and open areas (OAs) from 2015 to 2018.

Supplementary Figure 2 Principal Component Analysis (PCA) for the variance in leaf traits among different species and treatments. The horizontal axis represents the first principal component (PC1); the vertical axis represents the second principal component (PC2). E: *Elymus nutans*, P: *Potentilla anserina*, *P*_n_: net photosynthetic rate, *T*_r_: transpiration rate, *g*_s_: stomatal conductance, LUE: light use efficiency, *F*_v_/*F*_m_: maximum photochemical efficiency of PSII, *yield*: effective photochemical efficiency, *q*P: photochemical quenching, *q*N: non-photochemical quenching (*q*N), TC: total carbon content, TN: total nitrogen content, TP: total phosphorus contents, NP: the N:P ratios. TWC: the total water content, RWC: relative water content, LDMC: leaf dry matter content, SLA: specific leaf area, SLV: specific leaf volume.
