# Supplementary material for: Warming affects leaf light use efficiency and functional traits in alpine plants: evidence from a 4-year in-situ field experiment
Source: Front Plant Sci. 2024 Mar 19;15:1353762. doi: 10.3389/fpls.2024.1353762 (PMC10985207; doi:10.3389/fpls.2024.1353762)
Supplement: Supplementary file 1 [file Table_1.docx]

**Table S1** Comparative analysis of leaf photosynthetic characteristics, chlorophyll fluorescence characteristics, and leaf element content between *E. nutans* and *P. anserina* under different warming years. ** indicates significant differences among different years, species and treatments at the level of *p* < 0.05, and ns indicates not significant. *P*_n_: net photosynthetic rate, *T*_r_: transpiration rate, *g*_s_: stomatal conductance, LUE: light use efficiency, *F*_v_/*F*_m_: maximum photochemical efficiency of PSII, *yield*: effective photochemical efficiency, *q*P: photochemical quenching, *q*N: non-photochemical quenching (*q*N), TC: total carbon content, TN: total nitrogen content, TP: total phosphorus contents, NP: the N:P ratios.

| Significance | *P*_n_ | *T*_r_ | *g*_s_ | LUE | *F*_v_/*F*_m_ | *Yield* | *qP* | *qN* | TC | TN | TP | NP |
| --- | --- | --- | --- | --- | --- | --- | --- | --- | --- | --- | --- | --- |
| years | ** | ** | ** | ** | ** | ** | ns | ns | ** | ns | ** | ** |
| treatments | ns | ns | ns | ** | ** | ns | ** | ns | ns | ** | ** | ** |
| species | ** | ** | ** | ns | ** | ** | ns | ** | ** | ns | ns | ns |
| years* treatments | ns | ns | ns | ** | ** | ns | ns | ns | ns | ** | ns | ns |
| years*species | ns | ** | ** | ns | ** | ** | ns | ns | ns | ns | ns | ns |
| treatments*species | ns | ns | ns | ns | ns | ns | ns | ns | ns | ns | ns | ns |
| years*species* treatments | ** | ** | ns | ** | ** | ns | ns | ns | ns | ns | ns | ** |
