# Supplementary figures and images for: Warming affects leaf light use efficiency and functional traits in alpine plants: evidence from a 4-year in-situ field experiment
Source: Front Plant Sci. 2024 Mar 19;15:1353762. doi: 10.3389/fpls.2024.1353762 (PMC10985207; doi:10.3389/fpls.2024.1353762)

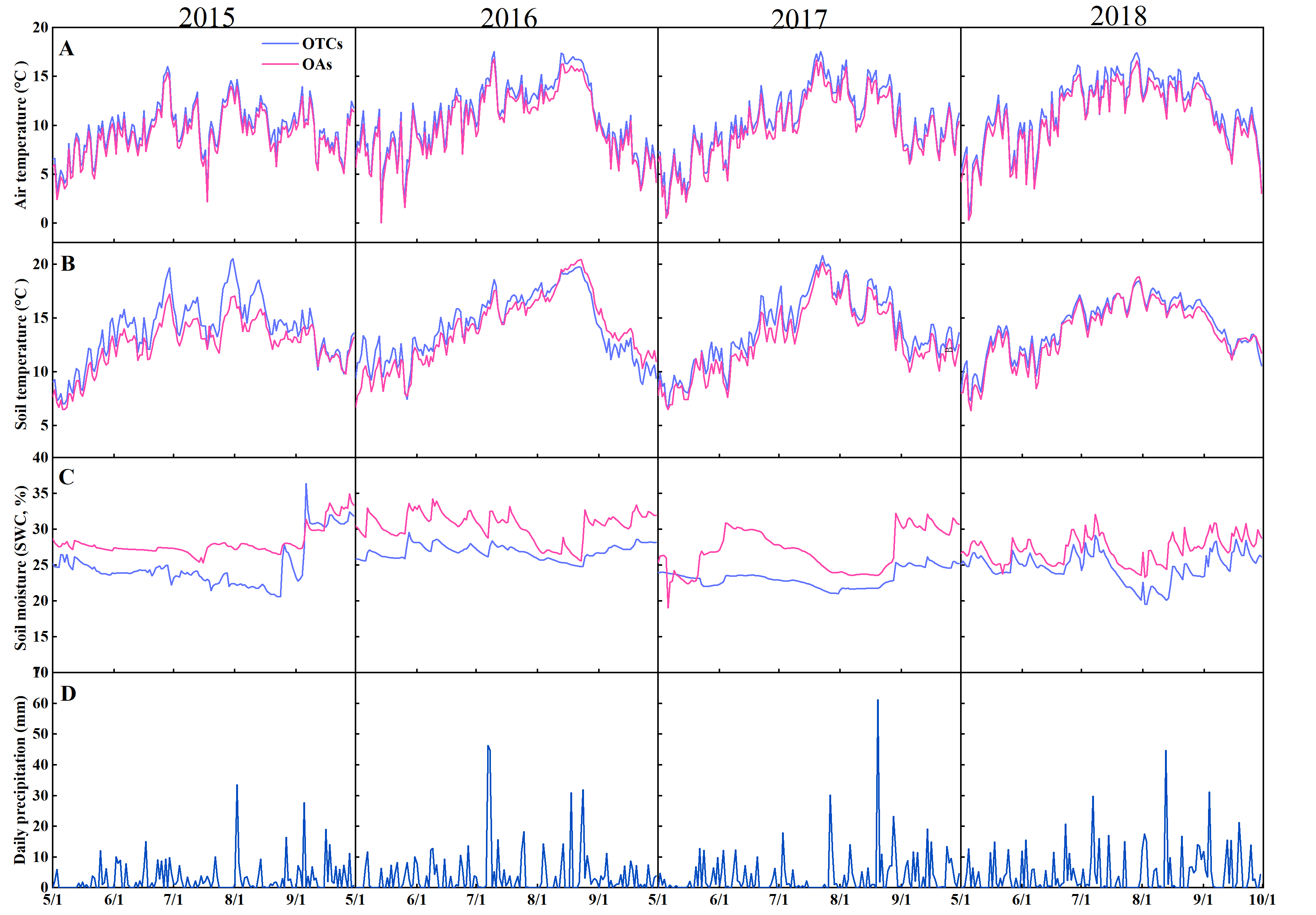

Supplement: Supplementary Figure 1 — Air temperature at a height of 1.5 m (°C), soil temperature at 5 cm depth (°C), soil moisture at 5 cm depth (%), and daily precipitation (mm) during the growing season (from May to September) in open-top chambers (OTCs) and open areas (OAs) from 2015 to 2018. [file Image_1.tif]

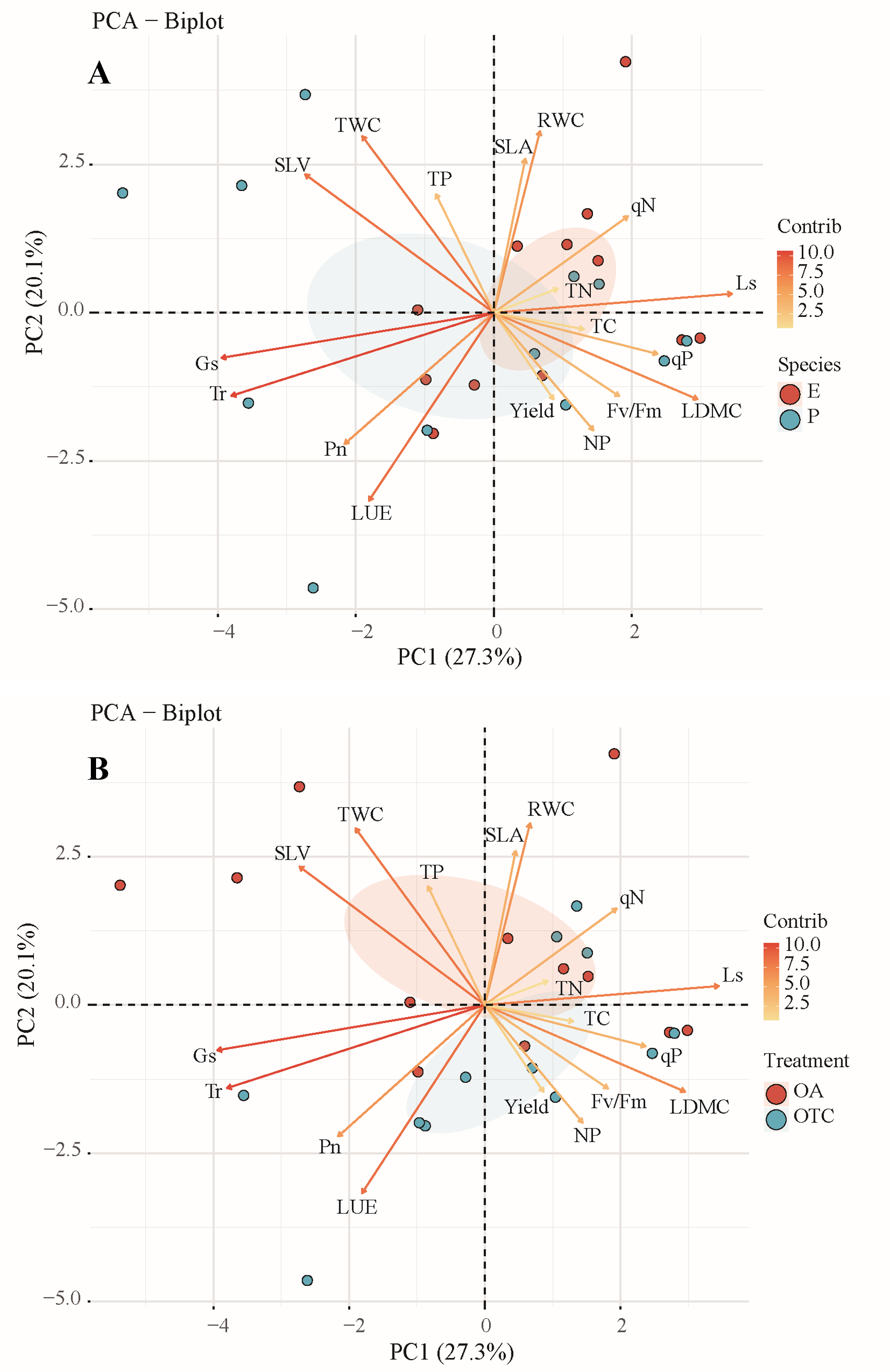

Supplement: Supplementary Figure 2 — Principal Component Analysis (PCA) for the variance in leaf traits among different species and treatments. The horizontal axis represents the first principal component (PC1); the vertical axis represents the second principal component (PC2). E: Elymus nutans, P: Potentilla anserina, P n: net photosynthetic rate, T r: transpiration rate, g s: stomatal conductance, LUE: light use efficiency, F v/F m: maximum photochemical efficiency of PSII, yield: effective photochemical efficiency, qP: photochemical quenching, qN: non-photochemical quenching (qN), TC: total carbon content, TN: total nitrogen content, TP: total phosphorus contents, NP: the N:P ratios. TWC: the total water content, RWC: relative water content, LDMC: leaf dry matter content, SLA: specific leaf area, SLV: specific leaf volume. [file Image_2.tif]
